# Far‐Red Light Supplementation in Tomato Cultivation: A Quantitative Review of Morphological and Physiological Responses

**DOI:** 10.1111/ppl.71141

**Published:** 2026-10-02

**Authors:** Anna Fischer, Irene Vänninen, Kristiina Himanen

**Affiliations:** ^1^ Department of Organismal and Evolutionary Biology University of Helsinki Helsinki Finland; ^2^ Natural Resources Institute Finland (Luke) Helsinki Finland; ^3^ National Plant Phenotyping Infrastructure, Helsinki Institute of Life Science (HiLife), Biocenter Finland University of Helsinki Helsinki Finland

## Abstract

Far‐red (FR) light at wavelengths 700–800 nm is abundant in sunlight but largely absent from artificial light sources. Standard Light‐Emitting Diodes emit little to no FR light. Recently, research has focused on the effects of FR light on the growth and development of crops, like tomatoes. However, findings on FR effects on tomatoes vary, with some effects even depending on the geographic location. To unravel the variability of the results, this review identified all relevant studies that quantified the FR‐induced responses in tomato growth, morphology, fruit development and photosynthetic parameters. The studies were categorised into controlled‐environment studies with artificial lighting (23 studies) and semi‐controlled studies with mixed artificial and sunlight (20 studies). To allow comparison between the FR treatments, three categories were established representing the given FR proportions at shade, sunlight or artificial levels. While FR responses were often cultivar‐dependent, known responses were confirmed for several parameters. Supplemental FR light increased plant height and often enhanced fresh weight, dry weight and leaf area, while stem diameter showed a decreasing response to FR. FR typically reduced leaf chlorophyll content, but its effects on photosynthetic efficiency varied. Importantly, variability in results appeared to arise from factors related to experimental setups rather than from confirmed FR quantities. To our knowledge, this is the first review providing a deep comparative analysis of FR treatments and experimental conditions in relation to tomato responses. This knowledge is crucial for planning controlled‐environment lighting strategies and will greatly facilitate and increase the value of future studies on tomato performance.

## 1 Introduction

Light is an essential environmental factor in the plant life cycle, regulating both growth and development. In recent years, there has been an increasing interest in the effects of far‐red (FR) light on plants, driven by the rising popularity of light‐emitting diodes (LEDs) in horticulture (Ji et al. 2021). LEDs have become popular due to their energy efficiency, low heat emissions and safety. Unlike other artificial light sources, LEDs allow for precise adjustment of the light spectrum (Morrow 2008) and thus enable detailed studies on FR light (Ji et al. 2021). Photons between 400 and 700 nm drive photosynthesis and are therefore described as photosynthetically active radiation (PAR) (Zhen et al. 2021). Photons above 700 nm up to 750 nm (Zhen et al. 2021) or 800 nm (Wassenaar et al. 2022) are called FR radiation. These wavelengths barely contribute to photosynthesis when applied alone and have therefore been excluded from the definition of PAR (Zhen et al. 2021). However, it has long been known that FR and PAR photons act synergistically to drive photosynthesis when applied together (Emerson and Lewis 1943). This effect can be explained by the distinct excitation spectra of PSI and PSII. While photons above 680 nm mainly excite PSI, lower‐wavelength photons primarily excite PSII (Duysens and Amesz 1962). Because FR photons drive photosynthesis as efficiently as PAR photons when applied together, it has recently been proposed that FR photons should be included in the definition of PAR. To distinguish the proposed new definition of PAR from the traditional one, the term extended PAR (ePAR) has been introduced for photons between 400 and 750 nm (Zhen et al. 2021).

Plants constantly monitor their light environment and perceive signals about it through different photoreceptors. One of them is phytochrome, a soluble protein encoded by a small number of multigenes. It exists in varying genetic types in higher plants. Phytochrome A and B play a crucial role in monitoring red (650 nm) and FR (750 nm) light (Nagatani 2004). The tomato genome encodes five phytochromes called PHYA, PHYB1, PHYB2, PHYE and PHYF (Hauser et al. 1995). Phytochromes generally exist in two photoconvertible forms: the biologically inactive red‐light absorbing form (Pr) and the biologically active far‐red‐light absorbing form (Pfr) (Nagatani 2004; Chen et al. 2005). Upon exposure to red light, Pr is transformed into Pfr. Upon exposure to FR light, Pfr is transformed into Pr (Casal 2000). Phytochromes control many developmental processes in plants through transcriptional regulation, from germination to stem elongation and leaf expansion to flowering. They also perceive neighbouring plants and shade (Neff et al. 2000). An increasing fraction of FR light in the light spectrum relative to red light triggers shade avoidance responses in plants as a survival mechanism to reduce the degree of shade for the plant. Some of the most common shade avoidance responses are stem elongation, a more erect leaf position and early flowering (Casal 2012). Such alterations in plant development can also be utilised in agricultural and horticultural production.

FR‐induced shade avoidance responses vary between species (Demotes‐Mainard et al. 2016; Hwang et al. 2020; Kump 2020; Zhang et al. 2024) and can therefore not be generalised. This review focuses on studies that specifically address the effects of FR light on tomato plants in Controlled Environment Agriculture (CEA) production. Tomato is one of the world's most important crop species (Cammarano et al. 2022) and also serves as a model species for both basic and applied research (Wu et al. 2025). CEA is a method of plant cultivation where the grower has a certain level of control over the environmental conditions, such as lighting, temperature and humidity. This allows for precise adjustment of the environmental conditions to the needs of the selected crop and can enhance productivity, sustainability, resilience to climate change and food safety (Neo et al. 2022).

To optimise controlled environment lighting strategies for tomato cultivars, it is essential to understand the critical factors that affect tomato performance. The results of similar studies can often yield contradictory findings, which can be attributed to the significant variability in environmental conditions present in those studies. Therefore, the aim of this review was to identify and categorise the key environmental factors documented in the literature that influence the impact of FR light on growth, morphology, fruit development and photosynthesis of tomato plants.

In this review, existing knowledge gaps on FR impacts on tomato growth were identified. While there are other reviews focusing on FR light signalling (Shomali et al. 2025), the effects of FR light on various crop species (Demotes‐Mainard et al. 2016; Kump 2020; Zhang et al. 2024) and the influence of different LED light spectra on tomato plants (Appolloni et al. 2021), this review uniquely provides a comprehensive comparative analysis of FR effects specifically on tomato plants grown in CEA. It confirms the established tomato responses to FR and indicates the possible sources of variability in the responses. Special attention is given to the shortcomings in experimental reporting, such as missing information on the background environmental conditions.

## 2 Review Method

Literature search was conducted across the databases Google Scholar, PubMed and Scopus. The search prompt was ‘tomato AND far‐red’, and the publication date was limited to 2010–2025 because there is a notable increase in relevant studies from 2010 onwards. Moreover, the research methods and reporting in earlier publications often differ from those in more recent studies. For Scopus, the prompt was limited to title, abstract and keywords. Additionally, several articles were identified through the references of relevant articles. To uncover potentially missed articles, several articles were transferred to the Keenious Plus platform, where the prompt ‘tomato AND far‐red’ was applied to the search results.

The titles and abstracts of potentially relevant articles were reviewed. If the abstract stated that FR light was applied to tomato plants, the article's methods section was analysed to ensure it met the predefined selection criteria listed in Table 1.

**TABLE 1:** Selection criteria for articles to be included in the review.

	Selection criteria
1	Peer‐reviewed article published in a scientific journal
2	The whole text is available in English
3	Published between 2010 and 2025
4	Experiments were conducted on tomato plants
5	Study includes at least one experimental treatment with far‐red light as the only varying factor and an appropriate control
6	The far‐red treatment was conducted during the cultivation period and not post‐harvest
7	The far‐red treatment was applied to the whole plant and not only to a specific plant part
8	The amount of far‐red light in the study is quantified in a way that makes the study reproducible
9	The parameters investigated are either plant growth, morphology, photosynthesis, fruit growth or quality, or a mix of these
10	Data from one study does not overlap with data from another study

If an article met the above‐described criteria and reported the results alongside a statistical analysis, it was included in the review. Statistically significant findings regarding plant growth, morphology, fruit development and photosynthetic parameters were extracted. However, the studies were often not comparable because growth and light conditions, as well as plant developmental stage, varied significantly. Therefore, the experimental conditions related to lighting, growth conditions and plant cultivar and age (Figure 1) were recorded for each study. The recorded factors included light conditions (photoperiod, timing of supplemental far‐red light during or after the photoperiod, lamp type, light intensity of far‐red and background light, light spectrum of far‐red and background light, and lamp position), growth conditions (temperature, humidity and CO_2_ concentration) and plant developmental factors (tomato cultivar, plant age at the start of the experiment and experimental length). These data are listed in Table S1a–c, which separately report the plant responses.

**FIGURE 1:**
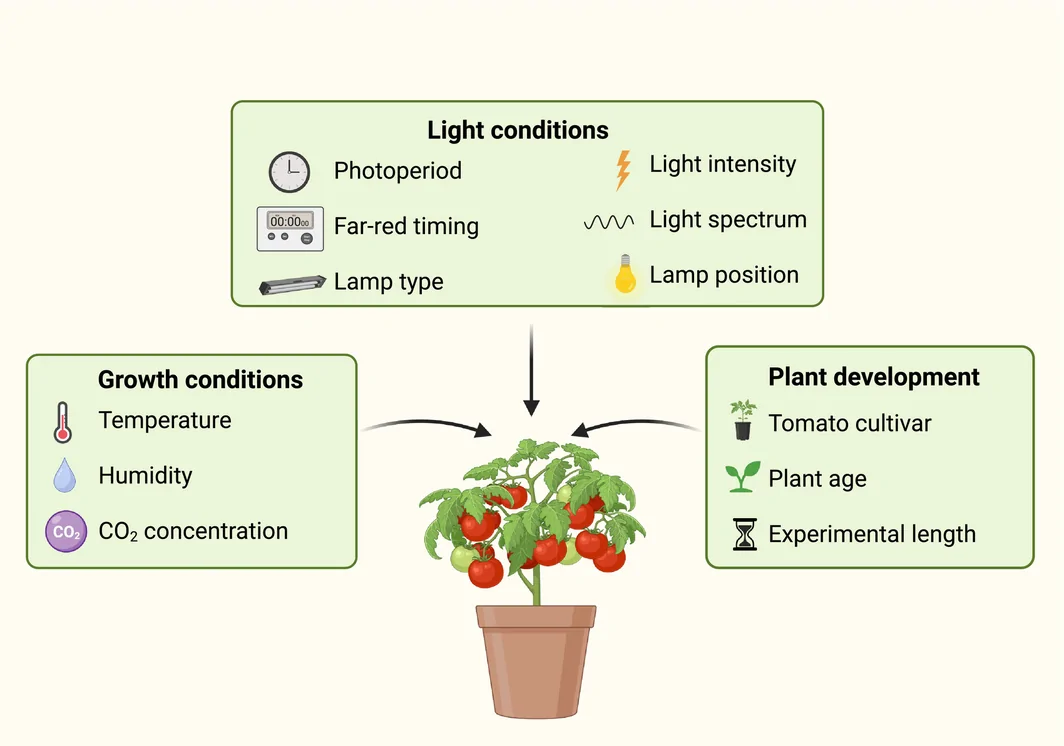
Varying environmental factors in the analysed studies. Environmental and plant developmental factors in the analysed studies were recorded alongside the findings. The recorded factors included light conditions (photoperiod, timing of supplemental far‐red light during or after the photoperiod, lamp type, light intensity of far‐red and background light, light spectrum of far‐red and background light, and lamp position), growth conditions (temperature, humidity and CO_2_ concentration) and plant developmental factors (tomato cultivar, plant age at the start of the experiment and experimental length). Created in BioRender. Fischer, A. (2026) https://BioRender.com/xyqm42r.

To allow for meaningful analysis of the environmental parameters and plant responses, the studies were divided into two main categories: studies utilising only artificial light (controlled environments, 23 studies) and studies utilising a mix of artificial light and sunlight (semi‐controlled environments, 20 studies).

For controlled‐environment studies, the minimum number of studies examining a parameter to be included in the review was two. The total number of observations was 239 (Tables S2–S4). An observation was defined as a comparison between two treatments, either both with different amounts of FR light or one with FR light and one without FR light, serving as a control treatment. Different background lighting or different cultivars under otherwise equal conditions also counted as an individual observation. Details regarding environmental conditions and amounts of supplemented FR light are reported in Table S1a. The number of studies and observations examining each parameter, the detected effects of FR light on these parameters, the magnitude of change in per cent, and the corresponding references are specified in Table S2 for growth and morphology, Table S3 for reproductive growth and Table S4 for photosynthetic efficiency. Magnitudes of change are visualised in Figure 2 for the most commonly studied parameters.

**FIGURE 2:**
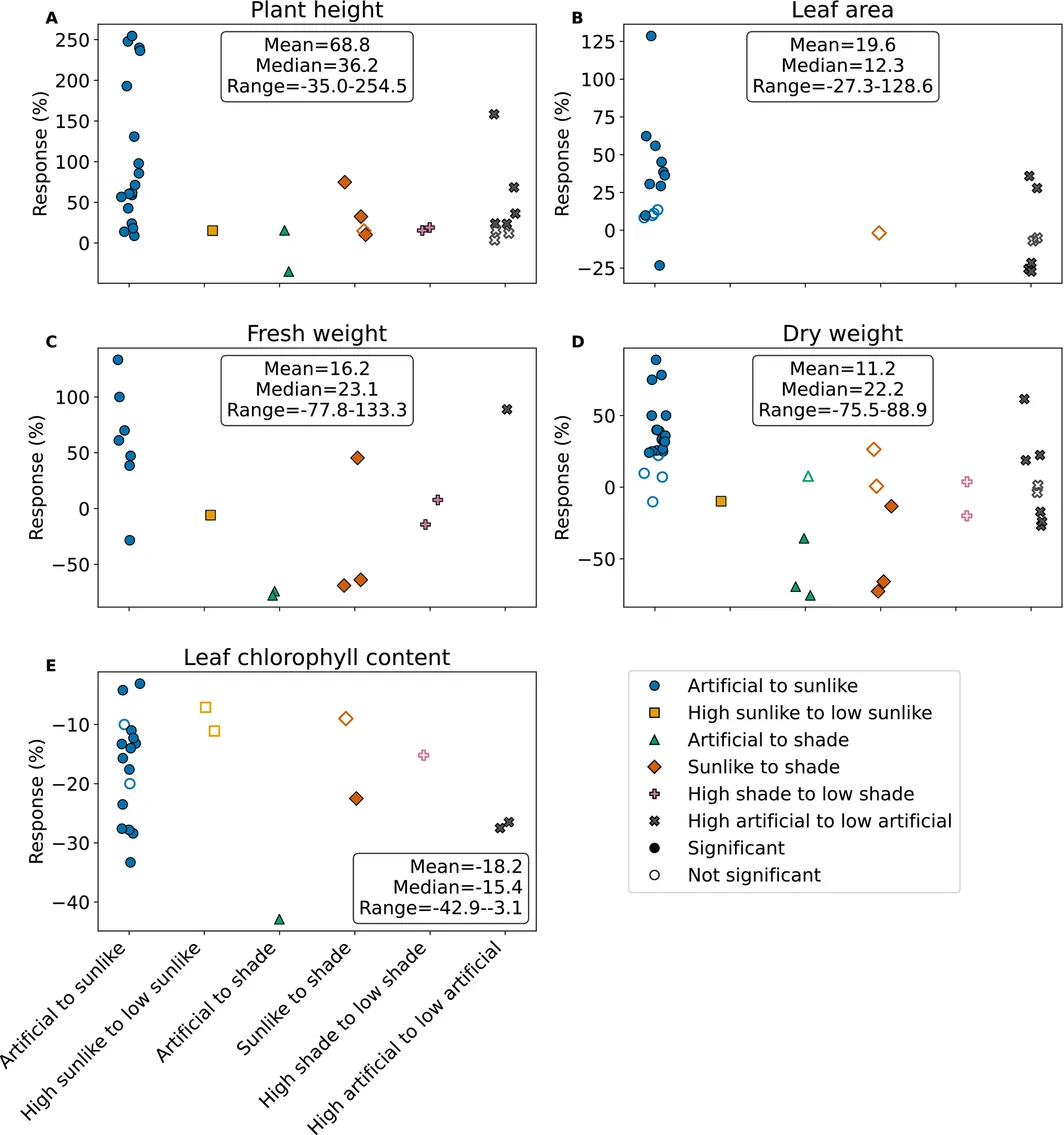
Magnitude of plant trait changes after far‐red supplementation. The five most commonly evaluated parameters across studies after far‐red supplementation in per cent are displayed: plant height (A, *n* = 35), leaf area (B, *n* = 22), fresh weight (C, *n* = 16), dry weight (D, *n* = 41) and leaf chlorophyll content (E, *n* = 24). Mean, median and range of responses in per cent are shown for each parameter. Each data point represents an individual observation in the respective category. The categories ‘artificial’, ‘sunlike’ and ‘shade’ represent far‐red proportions resembling artificial light sources, sunlight and shade, respectively.

For semi‐controlled studies, the amount of sunlight varied widely across studies due to differences in experimental setups, geographical locations, time of year and experimental duration. There were also significant fluctuations within a single experiment, making it almost impossible for researchers to accurately measure the amount of FR light a plant received. Comparisons across studies are therefore less robust for semi‐controlled environments, and no comparisons between single observations were made. The results are discussed separately from those of the controlled environment studies.

The studies also showed a wide range of FR intensities applied in the treatments. Therefore, for the controlled‐environment studies, the FR treatments were classified into three main categories as described in Table 2: FR light resembling artificial light sources (‘artificial’), sunlight (‘sunlike’) and shade (‘shade’). Additionally, if a study compared two or more treatments within the same category, this comparison was also included, provided that a statistical comparison was conducted and the results were reported accordingly.

**TABLE 2:** Proportion of FR light used in tomato studies. Four different measurement methods are used to quantify FR light, and categorisation of the level of FR light resembles ‘shade’, ‘sunlike’ and ‘artificial’ light.

Measures to quantify far‐red light	Shade	Sunlike	Artificial
Red to far‐red ratio (R:FR ratio)	< 0.69	0.69–1.6	> 1.6
Far‐red fraction (FR fraction)	> 0.60	0.60–0.38	< 0.38
Phytochrome Photostationary State (PSS)	< 0.67	0.67–0.73	> 0.73
Far‐red percentage (FR %)	> 19	17.1–19	< 17.1

## 3 Quantifying Far‐Red Light

The amount of FR light in white light depends on the light source. While sunlight naturally contains FR light, artificial light sources do so to a much lesser extent. An increasingly adopted artificial light source is LEDs. White LEDs emit practically no FR light (Wassenaar et al. 2022), but the spectrum can be expanded with additional channels. Another lamp type commonly used in greenhouses is the high‐pressure sodium (HPS) lamp, which typically emits only a small fraction of FR light (Hogewoning et al. 2010). White, red and blue LEDs were the most commonly used artificial light sources in the reviewed studies, followed by HPS lamps. Due to these differences in light sources and to allow comparisons between studies, it is crucial to accurately measure and report the amount of FR light in the supplemented light spectrum. The most common FR quantification method is the ratio between red and FR light; other options include the FR fraction, the Phytochrome Photostationary State and the amount of FR light expressed as a percentage. All these quantification methods provide a range of FR light in the provided light spectrum and reflect the different quantities and proportions of FR light to red or white light. Therefore, in this review three distinct categories for the quantification of FR light were established based on the FR light proportions resembling ‘sunlike’, ‘shade’ and ‘artificial’, where ‘sunlike’ matches the typical FR proportions in sunlight, ‘shade’ matches the high proportions of FR light observed in shade and ‘artificial’ matches low proportions obtained from artificial light sources. These newly established categories and their respective quantifications are presented in Table S1.

### 3.1 Red to Far‐Red Ratio

The most commonly used measure to quantify the amount of FR light in the light spectrum in the presented studies was the ratio between red and FR light (R:FR ratio). It is calculated by dividing the photon flux of red light by the photon flux of FR light (Kusuma and Bugbee 2021). When comparing R:FR ratios across studies, it is important to note that the definitions of red and FR wavebands vary between experiments. The most popular definition was made by Smith (1982), who defined red radiation as 655–665 nm for standard calculations and as 655–675 nm for simulated irradiances, and FR radiation as 725–735 nm. Hughes et al. (1984) and Chiang et al. (2019) supported the definition for standard calculations. Other authors proposed a narrower definition, such as 660 and 730 nm (Salisbury 1981) or a broader definition, such as 600–700 and 700–800 nm (Goldberg and Klein 1977), 600–700 and 725–735 nm (Lee and Downum 1991) for red and FR radiation, respectively.

The R:FR ratio of sunlight underlies significant diurnal and seasonal changes, both within a single geographical location and between different locations. Factors that influence these fluctuations include solar elevation angles, atmospheric water vapour and ozone levels (Kotilainen et al. 2020). In general, the R:FR ratio decreases during twilight due to low solar elevation angles when the sun is below the horizon (Kotilainen et al. 2020) and in shaded conditions (Mizuno et al. 2015). Several researchers measured the R:FR ratio of direct sunlight on different locations on Earth, namely Finland; India (Kotilainen et al. 2020); England (Holmes and Smith 1977; Smith 1982, 1994); Utah, USA (Salisbury 1981); Huntington, USA (Johnson et al. 2016) and Indiana, USA (Kim et al. 2019). The measured ratios varied due to the aforementioned factors and the fact that different wavelengths were used in the studies, resulting in ratios ranging from 0.69 to 1.6. Therefore, red to FR ratios between 0.69 and 1.6 are considered ‘sunlike’. Lower R:FR ratios are considered ‘shade’, and higher ratios are considered ‘artificial’.

### 3.2 Far‐Red Fraction

A recently developed method to quantify the fraction of FR light in the spectrum and phytochrome effects on plant morphology is the FR fraction (Kusuma and Bugbee 2021). The authors suggested using the FR fraction as an improved metric over the R:FR ratio. The FR fraction is calculated using the formula [FR/(R + FR)], where R refers to red light and FR refers to far‐red light. The authors suggested using a range of 730 ± 10 nm for FR light. The FR fraction ranges from 0 to 1. Values closer to 0 indicate a smaller amount of FR light in the spectrum. Using the given equation and the R:FR ratios of sunlight as stated in Section 3.1, typical values for the FR fraction of sunlight range from 0.60 to 0.38. Thus, in this review, FR fractions between 0.60 and 0.38 are considered ‘sunlike’. Higher fractions are considered ‘shade’, and lower fractions are considered ‘artificial’ (Table 2).

### 3.3 Phytochrome Photostationary State

Another method to bypass the problem of R:FR ratios approaching infinity is using the Phytochrome Photostationary State (PSS). The PSS includes all photons from 300 to 800 nm and describes the ratio of the biologically active Pfr to the total of Pr and Pfr (Sager et al. 1988). It can be calculated from the spectral distribution data of a light source as follows:

$$\;\frac{\sum_{300}^{800}N\left(\lambda \right)\sigma \left(r\lambda \right)}{\left(\sum_{300}^{800}N\left(\lambda \right)\sigma \left(r\lambda \right)+\sum_{300}^{800}N\left(\lambda \right)\sigma \left(\mathrm{fr}\lambda \right)\right)}$$

where *N*(*λ*) indicates the photon flux, and *σ*(r*λ*) and *σ*(frλ) indicate the photochemical cross‐section of Pr and Pfr, respectively (Sager et al. 1988; Hwang et al. 2020). The PSS of sunlight ranges from 0.67 to 0.73 (Sager et al. 1988; Kalaitzoglou et al. 2019; Jishi 2024; Lazzarin et al. 2024). Jishi (2024) reports PSS values of approximately 0.9 for red LED light, 0.8 for green LED light, 0.5 for blue LED light and 0.1 for FR LED light. In this review, PSS values of 0.67–0.73 are considered ‘sunlike’. Lower PSS values are considered ‘shade’, and higher values are considered ‘artificial’ (Table 2).

### 3.4 Far‐Red Percentage

The percentage of FR light in sunlight varies, but typically ranges from 17.1% to 19% relative to PAR (Zhen and Bugbee 2020; Zhen et al. 2022). In vegetation shade, sunlight contains 81%–93% FR light relative to PAR (Zhen et al. 2022). In this review, FR percentages between 17.1% and 19% relative to PAR are considered ‘sunlike’, higher percentages are considered ‘shade’, and lower percentages are considered ‘artificial’ (Table 2).

## 4 Effects of Far‐Red Light in Controlled Environments

Here, literature on the effects of supplemental FR light on tomato growth and morphology, fruit development and quality, and photosynthesis in controlled environments without sunlight is reviewed. This analysis allows for the establishment of the magnitude and range of change. After the main findings for each parameter, examples of studies deviating from the most typical responses are highlighted. Detailed information on experimental conditions, light treatments, lamp position, FR amount and wavelength, and changes in the measured parameters can be found in Table S1a (growth and morphology), Table S1b (reproductive growth) and Table S1c (photosynthetic efficiency), the newly established categories of FR light proportions representing artificial, sunlike and shade conditions (Table 2).

### 4.1 Effects on Plant Growth and Morphology

#### 4.1.1 Effects on Plant Height

Plant height growth is mediated by cell division, cell elongation, internode elongation and ultimately also by increasing the number of nodes. Fifteen studies with 36 observations examined the effects of FR light on tomato plant height. All 15 studies, comprising 31 observations (86.1%), found a significant increase in plant height upon FR exposure. The overall response magnitude ranged from −35% to +254.5%, with a median response of +36.2% and a mean response of +68.8%. The strongest increase was achieved when FR levels were raised from artificial to sunlike levels or to artificial but close to sunlike levels (‘low artificial’, Figure 2A, Table S2). The growth‐enhancing effect of FR light was detected across different environmental conditions (such as varying temperatures, humidity levels, photoperiods and CO_2_ concentrations), different amounts of supplemental FR light (ranging from ‘shade’ to ‘artificial’) and different background light intensities and spectra (Table S1a).Even though most studies were conducted using tomato seedlings, the growth‐enhancing effect was also observed in mature tomato plants (Miao et al. 2024).

Only two studies with four observations (11.1%) found no significant effect. Non‐significant FR effects seemed to be explained by only minor changes in the amount of FR light or by temperature. Jeong et al. (2024) reported three observations with no significant effect. In this study, three of six observations of plant height increased, and three showed no significant effect; the only difference between the treatments was temperature. At 28°C, adding 25 μmol of FR light to a background of 250 μmol led to a significant increase in plant height, whereas at 22°C, it showed a similar but not significant trend. The authors found a significant interaction between FR light and warmer temperatures, suggesting a synergistic effect.

Also, the one study with two observations (5.6%) that found a significant decrease in plant height (Gai et al. 2022) relates to the quantification of FR supplement. The authors found the effects of FR to be dependent on the background light spectrum. While supplemental FR light increased plant height under a combination of red and blue background lighting, it caused a decrease in plant height under white light. On the other hand, the amount of supplemented FR light differed between these two treatments and was much higher under white background light (25%) than under red and blue light (~14%). In combination with the pretty low background light intensity (100 μmol), supplementing such a high percentage of FR light might have been too much to support optimal plant growth.

#### 4.1.2 Effects on Hypocotyl Length

Hypocotyl elongation is a mechanism by which seedlings emerge from the soil. After emergence, in non‐optimal light, hypocotyls continue elongating. In controlled environments, FR light had varying effects on hypocotyl growth. Three studies, with 11 observations, examined the effects of FR light on hypocotyl length. All three studies with five observations (45.5%) found a significant increase in hypocotyl growth upon FR exposure, two studies with four observations (36.4%) found no significant effects and one study with two observations (18.2%) detected a significant decrease. Response magnitudes ranged from −20.6% to +39.5%, with a median response of +8.6% and a mean response of +7.4% (Table S2). Environmental conditions and cultivar selection varied widely across these studies. While Hwang et al. (2020) found that sunlike FR amounts had the most enhancing effect, Ma and Upadhyaya (2016) and Li et al. (2025) found a significant increase when supplementing amounts of FR light that resembled strong shade. Therefore, the responses of hypocotyl length to FR light could have been cultivar‐dependent, with mostly an enhancing or neutral effect.

#### 4.1.3 Effects on Leaf Area

Regarding tomato leaf area, of nine studies with 23 observations, eight studies with 12 observations (52.2%) found a significant increase upon FR exposure; four studies with seven observations (30.4%) found no significant effect; and two studies with four observations (17.4%) found a significant decrease. The magnitude of change showed a big variation from −27.3% to +128.6%, with a median of +12.4% and a mean of +19.6% (Table S2). Aiming for sunlike FR levels caused the strongest increase across studies (Figure 2B), even under a low background light intensity (Wang et al. 2021).

In a study of six tomato cultivars, Retana‐Cordero et al. (2022) showed that leaf area response to FR light was cultivar‐dependent, with mostly enhancing or no effect. These observations were also supported by Ji et al. (2021). Another factor that seems to influence leaf area responses to FR light is the background light spectrum. Zhang et al. (2022) showed that the presence of green light in the background light spectrum reduced the effects of FR light on leaf area.

Similarly to plant height, Jeong et al. (2024) also reported mixed results for leaf area with a trend towards a greater decrease in leaf area upon FR exposure at lower temperatures (22°C) than at higher temperatures (28°C). FR supplementation had no significant effect on leaf area across six different cultivars from four studies (Kurepin et al. 2010; Retana‐Cordero et al. 2022; Zhang et al. 2022; Jeong et al. 2024).

#### 4.1.4 Effects on Fresh Weight

Supplemental FR light had varying effects on tomato fresh weight in the six examined studies with 16 observations. Five studies with nine observations (56.3%) found an increase in fresh weight upon FR exposure, while two studies with seven observations (43.8%) found a decrease. The magnitude of change varied a lot across studies, ranging from −77.8% to +133.3%, resulting in a median of +23.1% and a mean of +16.2% (Table S2). Aiming for sunlike FR levels consistently enhanced fresh weight, even under varying background light spectra (Park and Runkle 2023). Aiming for shade‐like ratios either reduced it (Figure 2C) or only led to a small increase (Guo et al. 2024).

Six out of seven observations showed a reduction in fresh weight in one study (Hwang et al. 2020). Since the environmental conditions were comparable to those in other studies in this category, and this study was the only one using its respective cultivar (‘Dotaerangdia’), the observed decrease could have been due to cultivar‐dependent responses to FR light, similarly to Ji et al. (2021). The exact amount of FR light in the spectrum likely also played a role. Cao, Yu, et al. (2018) found an increase in fresh weight when reducing the R:FR ratio from ratios that can be considered ‘artificial’ to ratios that resemble sunlight. On the other hand, they found a decrease when slightly reducing the R:FR ratio while remaining within the ‘artificial’ range.

#### 4.1.5 Effects on Dry Weight

Tomato dry weights showed substantial variability in the 15 studies with 43 observations. Of these, 10 studies with 21 observations (48.8%) found a significant increase in dry weight upon FR exposure; 8 studies with 12 observations (27.9%) found no significant effect; and 5 studies with 10 observations (23.3%) found a decrease. The magnitude of change ranged from −75.5% to +88.9%. The median response was +22.2%, and the mean response was +11.2% (Table S2). Supplementing FR levels that resembled those of sunlike conditions almost universally increased dry weight, while aiming for shade or artificial levels often caused only a minor increase (Ji et al. 2023) or a decrease (Figure 2D). Several factors, including the duration of the experiment, cultivar selection, the developmental stage of the plants and the intensities of FR and background light, likely contributed to the observed differences.

Two of the nine observations found no significant effect (Zhou et al. 2023, 2025) were from studies with a very short duration of 5 days. This short duration could explain why no effects were detected. Courbier et al. (2021) found that FR light had either an enhancing or no significant effect on leaf dry weight, depending on the developmental stage of the measured leaves. Accordingly, leaves that had already developed prior to being exposed to FR light were less affected than leaves that were growing under FR light. While all environmental conditions in the three studies were similar, Courbier et al. (2021) reduced the R:FR ratio more than Zhou et al. (2023, 2025), resulting in light conditions resembling extreme shade. This difference in the amount of FR light likely explains the stronger response in the Courbier et al. (2021) study. Retana‐Cordero et al. (2022) reported one non‐significant observation alongside five significant ones, the only difference being the cultivar selection.

Similarly, the non‐significant findings of Ma and Upadhyaya (2016) and the mostly decreasing effects reported by Hwang et al. (2020) might have resulted from selecting weakly responding cultivars (‘Gold Nugget Cherry’, ‘Dotaerangdia’). The results of Johnson et al. (2016) indicated that FR amounts that resemble sunlight had the most enhancing effect on dry weight, followed by amounts resembling artificial light sources, while shade‐resembling amounts of FR light reduced dry weight. Cao, Yu, et al. (2018) obtained similar results with sunlike R:FR ratios resulting in a stronger response than a R:FR ratio that resembled artificial light. Shomali et al. (2024) showed an enhancing effect in high‐intensity background lighting and a reducing effect in low‐intensity background lighting. However, the R:FR ratios differed between these treatments.

#### 4.1.6 Effects on Stem Diameter

Five studies, comprising 15 observations, examined the effects of FR light on tomato stem diameter. In the reviewed studies, supplemental FR light mainly had either no or a reducing effect on stem diameter. One study with one observation (6.7%) found a significant increase in stem diameter upon FR exposure, four studies with seven observations (46.7%) found no significant effect and three studies with seven observations (46.7%) found a decrease. The magnitude of change ranged from −38.8% to +9.7%, with a median of −6.4% and a mean of −12.3% (Table S2). Light intensity and experimental duration varied widely across these studies, and no clear response pattern emerged from their comparison. Each study used a different cultivar, as far as the cultivar was specified. This difference may have contributed to the inconsistent responses.

### 4.2 Effects on Fruit Development and Quality

#### 4.2.1 Effects on Flowering Time

Flowering time is controlled through a complex regulatory network. It integrates both endogenous and external signals that promote the transition from the vegetative stage to reproduction. Flowering time can be defined by the number of leaves at bolting or by the number of days from germination to bolting (Pavicic et al. 2017).

Two studies, each with five observations, examined the effects of FR light on tomato flowering time. Of these, two studies with four observations (80%) found significantly delayed flowering, and one study with one observation (20%) found an accelerated flowering time. The magnitude of change ranged from −17.9% to +50%, with a median of +26.3% and a mean of +22.9% (Table S3).

Shomali et al. (2024) found that FR light led to earlier flowering under high background light intensities and delayed flowering under low background light intensities. Cao, Yan, et al. (2018) report delayed flowering under FR exposure in controlled environments. Under FR exposure, the plants had more leaves when flowering. No other parameter to measure the flowering time, such as the day after germination or transplant, was recorded in this study. Since several studies have linked FR supplementation to increased leaf numbers (Ji et al. 2019; Kim and Hwang 2019), the leaf stage may not be an accurate indicator of FR light impacts on flowering time for tomato plants.

Two studies with two observations evaluated the effects of FR light on the glucose and fructose content of ripe fruits; one found an increase, and the other found no significant effect. Change magnitudes ranged from +7.7% to +37.1%, with a median and mean of +22.4% (Table S3). Both studies found an increase, even though only significant in one case, suggesting that FR supplementation increased the fruits' sugar content.

### 4.3 Effects on Photosynthesis

The role of FR in photosynthesis is related to its ability to excite PSI, and it participates in PAR. Photosynthesis mediates plant growth through the process of light harvesting for energy and the generation of organic molecules from CO_2_ (Stirbet et al. 2020). Chlorophyll is a pigment in the light‐harvesting complexes, mainly in forms of chlorophyll *a* and *b* (Hoober et al. 2007). Leaf chlorophyll content can be analysed by biochemical assays or transmittance‐based or fluorescence‐based portable chlorophyll meters (Lhotáková et al. 2025). The maximum and effective quantum yield of PSII are essential parameters to evaluate photosynthetic efficiency, widely used to assess plant fitness. Effective quantum yield is measured during constant illumination, and both parameters can be measured with stationary or handheld devices using pulse‐amplitude modulated (PAM) fluorimetry or Joliot‐type spectrometers (Chukhutsina et al. 2018). The net photosynthesis rate, the net rate of carbon fixation, is an indicator of overall plant health and functionality and serves as an early indicator of plant stress (Kalaji et al. 2016).

#### 4.3.1 Effects on Leaf Chlorophyll Content

Seven studies with 24 observations examined the effects of FR light on leaf chlorophyll content. Out of these, two studies with five observations (20.8%) found no significant effect, and seven studies with 19 observations (79.2%) found a significant decrease. The magnitude of responses ranged from −42.9% to −3.1%, with a median of −15.5% and a mean of −18.2% (Table S4). Most non‐significant observations could be explained by relatively low levels of supplemented FR light (Figure 2E).

#### 4.3.2 Effects on Maximum Quantum Yield of PSII

Three studies with nine observations examined the effects of FR light on the maximum quantum yield of PSII. Out of these, one study with one observation (11.1%) found a significant increase upon FR exposure, and three studies with eight observations (88.9%) found no significant effect, suggesting no interaction between FR and PSII. Change magnitudes ranged from ±0% to +1.9%, with a median of +0.7% and a mean of 0.9% (Table S4).

#### 4.3.3 Effects on Effective Quantum Yield of PSII

Three studies with 11 observations examined the effects of FR light on effective quantum yield of PSII. Out of these, two studies with five observations (45.5%) found a significant increase upon FR exposure, one study with one observation (9.1%) found no significant effect and two studies with five observations (45.5%) found a significant decrease. The three observations finding the strongest decrease by Cao, Yu, et al. (2018) had relatively low background light intensity compared to the other studies. The magnitude of change ranged from −14.3% to +20.9%, with a median of −1.7% and a mean of +1.7% (Table S4).

#### 4.3.4 Effects on Net Photosynthesis Rate

Four studies with nine observations examined the effects of FR light on net photosynthesis rate. Of these, two studies with one observation each found an increase or no significant effect (11.1%), and two studies with six observations (66.7%) recorded a decrease. Change magnitudes ranged from −37.1% to +14.9%, with a median of −17.8% and a mean of −12.6% (Table S4).

## 5 Effects of Far‐Red Light in Semi‐Controlled Environments

The chapter on semi‐controlled growth conditions examines the effects of supplemental FR light on plant growth and morphology, fruit development and quality, and photosynthesis in studies conducted in greenhouse conditions. In these conditions, plants received a part of the light spectrum from sunlight.

### 5.1 Effects on Plant Growth and Morphology

In semi‐controlled environments, FR light had effects similar to those in controlled environments. Even though the environmental conditions varied significantly between studies, FR light almost universally increased plant height (Hao et al. 2016; Johnson et al. 2016; Kim and Hwang 2019; Kalaitzoglou et al. 2019; Zhang et al. 2019; Zhou et al. 2021; Saha et al. 2022; Meijer et al. 2023; Vincenzi et al. 2025). Only very few studies found no significant effect (Kim et al. 2019; Appolloni et al. 2022) or a decrease (Wei et al. 2018; Jiang et al. 2018).

The growth‐enhancing effect of FR light was attributed to increased internode length rather than an increase in the number of nodes (Zhang et al. 2019). While Hao et al. (2016) reported that the effect of FR light was only present during the early growth stages and diminished over time, Meijer et al. (2023) reported that the effect of FR light increased over time. This difference might have been caused by seasonal changes and differences in the experimental design. In the two studies that found a decrease in plant height (Wei et al. 2018; Jiang et al. 2018), there was ample natural light, so that additional FR light might have shifted the spectral composition towards a higher FR fraction.

Supplemental FR light also increased leaf area in the majority of studies (Jiang et al. 2018; Kim and Hwang 2019; Kalaitzoglou et al. 2019; Kim et al. 2019). Vincenzi et al. (2025) found the effect of FR light on leaf area to be cultivar‐dependent, with either a slight increase or no effect, and Zhang et al. (2019) reported no significant effect. Zhang et al. (2019) used a relatively low background light intensity compared to the other studies, which might explain this observation. Vegetative fresh weight largely increased upon FR supplementation (Jiang et al. 2018; Kim and Hwang 2019; Zhang et al. 2019; Kim et al. 2020; Zhou et al. 2021; Saha et al. 2022). Only one study (Saha et al. 2022) reported no significant effect. The vegetative dry weight responded similarly with a significant increase in most studies (Jiang et al. 2018; Kim and Hwang 2019; Kalaitzoglou et al. 2019; Kim et al. 2019, 2020; Palmitessa et al. 2020; Zhou et al. 2021), only decreasing in one study (Wei et al. 2018). It was also reported that a PSS just above sunlike values had the most pronounced effect on vegetative dry weight (Kalaitzoglou et al. 2019).

There are contradictory results regarding stem diameter. While Jiang et al. (2018) and Zhou et al. (2021) reported a significant increase upon FR exposure, Kim and Hwang (2019) detected no significant effect and Wei et al. (2018) reported a decrease. These differences might be due to different cultivars or varying environmental conditions.

The number of leaves was mostly not significantly affected by FR light (Kalaitzoglou et al. 2019; Ji et al. 2019, 2020; Kim et al. 2019; Vincenzi et al. 2025). However, Kim and Hwang (2019) and Ji et al. (2019) reported significant increases, whereas Wei et al. (2018) reported a significant decrease. One interesting finding was that the observed effect depended on the background light. Ji et al. (2019) reported that FR light increased the leaf number when using a combination of sunlight, red and blue LEDs as background lighting, while there was no effect when using sunlight supplemented with white LEDs as background lighting.

### 5.2 Effects on Fruit Development and Quality

Overall, supplemental FR light had favourable effects on fruit development, but the findings varied across studies. The majority of studies reported an increase in yield or fruit fresh weight upon FR exposure (Hao et al. 2016; Zhang et al. 2019; Kalaitzoglou et al. 2019; Kim et al. 2019, 2020; Vincenzi et al. 2024). Vincenzi et al. (2025) reported a non‐linear increase, with the biggest increase at FR fractions of 0.40. Increasing the amount of FR light further had no effect or reduced fruit fresh weight again. However, some studies did not detect a significant effect (Palmitessa et al. 2020), found the effect to be dependent on the fruit cluster (Kim and Hwang 2019) or found it to be time‐dependent (Fanwoua et al. 2019). Similarly, FR light almost universally increased fruit dry weight (Ji et al. 2019; Zhang et al. 2019; Kim et al. 2019, 2020; Vincenzi et al. 2024, 2025). Ji et al. (2019, 2020) and Vincenzi et al. (2025) also detected an increase in the fraction of dry mass partitioned to fruits, while Zhang et al. (2019) reported a decrease. In some cases, FR light increased the number of fruits (Hao et al. 2016; Kalaitzoglou et al. 2019; Kim et al. 2019), but in other cases, there was no significant effect (Palmitessa et al. 2020; Vincenzi et al. 2024, 2025).

FR either accelerated flowering (Kim and Hwang 2019; Kim et al. 2019) or had no significant effect (Ji et al. 2020; Vincenzi et al. 2024). Time until harvest or fruit ripening time was significantly reduced in several studies (Kalaitzoglou et al. 2019; Kim et al. 2019, 2020; Ji et al. 2020; Meijer et al. 2023; Vincenzi et al. 2025), with only Vincenzi et al. (2024) finding no significant effect.

In some studies, FR light increased the fruit sugar content in ripe fruits (Fanwoua et al. 2019; Ji et al. 2020; Vincenzi et al. 2025), but other authors reported no significant effect (Vincenzi et al. 2024) or even a decrease (Zhang et al. 2019). Consumer panels either reported better sensory properties of tomatoes grown under FR light (Kim et al. 2020) or no difference (Dzakovich et al. 2017).

### 5.3 Effects on Photosynthesis

FR supplementation mostly decreased leaf chlorophyll content (Wei et al. 2018; Kim and Hwang 2019; Kalaitzoglou et al. 2019; Kim et al. 2019) or had no significant effect (Hao et al. 2016; Wei et al. 2018; Kim and Hwang 2019; Kalaitzoglou et al. 2019; Appolloni et al. 2022). Only one study reported an increase (Hao et al. 2016). This variation can likely be explained by the presence of natural FR light in semi‐controlled environments. Adding more FR light from artificial sources had no further effect in many cases. This effect can also be seen in single studies that supplement with varying amounts of FR light (Kalaitzoglou et al. 2019).

The effects of FR light on other photosynthetic parameters varied across the studies, and a relatively small number of studies even recorded these parameters. This can likely be explained by the considerable variation of light intensity and spectrum in greenhouses both throughout the day and throughout long experiments. While Jiang et al. (2018) found an increase in overall photosynthetic efficiency, Kim et al. (2019) and Palmitessa et al. (2020) reported no significant effect. Similarly, Vincenzi et al. (2025) found no significant effects of FR light on any photosynthetic parameter. Vincenzi et al. (2025) suggest that the lack of measurable effects in their own study may be explained by the overlap of two opposing FR effects: the short‐term enhancement effects through balancing the excitation of PSI and PSII (Emerson and Lewis 1943), and the reduced leaf chlorophyll content, which can affect photosynthetic efficiency negatively. The net photosynthesis rate was either reduced (Ji et al. 2019), unaffected (Vincenzi et al. 2024) or dose dependent (Kalaitzoglou et al. 2019). Zhou et al. (2021) reported an increase in the effective quantum yield of PSII, but the maximum quantum yield remained unchanged. Ji et al. (2019) found quantum yield to be generally dependent on the background lighting. When white light was part of the background light spectrum, quantum yield was not affected. However, compared to a background spectrum containing red and blue light, quantum yield was decreased.

### 5.4 Duration and Timing of Far‐Red Light

A potentially cost‐saving alternative to supplementing FR during the whole photoperiod could be to apply FR light only as a start‐of‐day or as an end‐of‐day FR (EOD‐FR) treatment. Growth parameters and fruit development were affected differently across previous studies, depending on whether FR was supplemented during or after the photoperiod.

Chia and Kubota (2010) found that an EOD‐FR treatment at the end of the photoperiod increased hypocotyl length of tomato seedlings by up to 40% compared to an untreated control. Similarly, Zhang et al. (2019) reported an EOD‐FR treatment to be as effective as a whole‐photoperiod treatment in promoting tomato plant growth and yield. Fruit sugar content was highest in the EOD‐FR treatment. In contrast, Vincenzi et al. (2024) found a linear correlation between FR duration and increased tomato fruit yield and weight. EOD‐FR treatments after the photoperiod, thus in the absence of PAR, proved to have only a marginal (Kalaitzoglou et al. 2019; Appolloni et al. 2022) or no effect (Appolloni et al. 2023) on tomato development. This can be explained by the fact that FR light is more effective when applied in combination with PAR photons (Emerson and Lewis 1943).

## 6 Future Perspectives on Improving Tomato Performance by Far‐Red Lighting

This review thoroughly examines the impact of far‐red (FR) light on tomato growth by deep analysis of relevant studies, not only for their results but also for the experimental setups in which they were conducted. As a result, the literature was categorised into controlled and semi‐controlled studies based on the FR light source—either fully controlled FR supplementation or a mixture derived from sunlight and artificial light. Research on FR responses indicates similarities across controlled and semi‐controlled (greenhouse) environments, although responses tend to be less pronounced in semi‐controlled settings due to the presence of natural FR radiation, which varies with time of day, season and geographic location.

Furthermore, FR lighting levels were classified into three categories: shade, sunlike and artificial to allow for comparison of the plant responses to the given FR supplement. Notably, both high and low FR light levels that mimic sunlight consistently improved growth metrics compared to those resembling artificial conditions, while high‐shade‐intensity FR lighting sometimes produced pronounced responses that did not always align with agricultural production goals. High‐shade‐intensity FR lighting promotes stem elongation but appears to inhibit secondary growth, resulting in reduced stem diameter and dry biomass, suggesting potential structural weaknesses in plants. Further molecular research would be needed to unravel the upstream regulatory pathways that influence cell wall biosynthesis.

The responses of tomatoes to FR lighting are influenced by numerous interacting environmental factors, creating complex relationships. However, the challenge in the current literature is the inconsistent reporting of environmental conditions. Most studies adhered to recommendations regarding supplemental FR light quantity, but the incomplete environmental context can hinder conclusions. This review is therefore also summarising the additional environmental factors that influence plant responses to FR lighting.

To facilitate reporting of supplemental FR light, the common methodologies for FR light quantification were summarised. From the four methods, the use of FR fraction or Phytochrome Photostationary State (PSS) appears more reliable over the R:FR ratio, which has limited standardisation and may yield erroneous extrapolations between artificial and natural light conditions.

Additionally, FR responses appear to be cultivar‐dependent, indicating the need for cultivar ranking to identify strong and weak responders. This information could guide future studies, helping to establish controls or test systems for comparative analysis and for selecting cultivars for the given purposes.

The review also highlights other observations that would require further investigation. Some studies hint that FR may enhance fruit sugar content, but more extensive research is required, particularly relevant for vertical farming systems, where, for example, dwarf tomato varieties are cultivated under fully controlled lighting.

An underexplored area is the interplay between FR light and light intensity, with evidence suggesting significant interactions. Studies have demonstrated that FR light can enhance biomass and photosynthetic efficiency under high‐light conditions but might be detrimental under low‐light situations. However, definitions of ‘high’ and ‘low’ light for tomatoes are unclear, necessitating further investigation into these interactions. Also, the role of FR in photosynthesis has not been revealed in the current literature, and the ePAR and photosynthetic role of FR wavelengths remain unclear.

In conclusion, while supplemental FR light has demonstrated potential to improve tomato growth and fruit quality, many interactions with environmental conditions remain inadequately understood and require further exploration. Nevertheless, the strategic use of FR lighting represents a promising approach to enhancing tomato performance in controlled environment agriculture (CEA).

## Author Contributions

**Anna Fischer:** conceptualisation, methodologies, visualisation, writing – original draft, writing – review and editing, project administration. **Irene Vänninen:** conceptualisation, writing – review and editing. **Kristiina Himanen:** conceptualisation, writing – review and editing, supervision.

## Supporting information


**Table S1:** Detailed information on experimental set ups in controlled conditions, including light treatments (lamp position, far‐red amount and wavelength), growth conditions (temperature, humidity and CO_2_ concentration) and plant developmental factors (tomato cultivar, plant age at the start of the experiment and experimental length). Changes as indicated as + for positive, 0 for neutral and − as negative responses are given in the separate worksheets for growth and morphology (S1a), reproductive growth (S1b) and photosynthetic efficiency (S1c). The magnitude of change in the given study is given as percentage under the categories of the level of FR light artificial/sunlike/shade and the direction of treatment change. Publication references are sorted by publication date starting from oldest.

**Table S2:** Parameters examining plant growth and morphology in controlled environments, number of studies and observations within those examining these parameters, detected effects of far‐red light on these parameters (‘+’ stands for a statistically significant increase upon FR exposure, ‘0’ for no statistically significant effect and ‘−’ for a statistically significant decrease), the magnitude of change in per cent and the corresponding studies by their reference number.
**Table S3:** Parameters examining reproductive growth in controlled environments, number of studies and observations examining these parameters, detected effects of adding far‐red light on these parameters (‘+’ stands for a statistically significant increase upon FR exposure, ‘0’ for no statistically significant effect and ‘−’ for a statistically significant decrease), the magnitude of change in per cent and the corresponding studies.
**Table S4:** Parameters examining photosynthesis of tomato plants in controlled environments, number of studies and observations examining these parameters, detected effects of adding far‐red light on these parameters (‘+’ stands for a statistically significant increase upon FR exposure, ‘0’ for no statistically significant effect and ‘−’ for a statistically significant decrease), the magnitude of change in per cent and the corresponding studies.

## Data Availability

The data that support the findings of this study are available in the Supporting Information of this article.
