# Supplementary material for: Far‐Red Light Supplementation in Tomato Cultivation: A Quantitative Review of Morphological and Physiological Responses
Source: Physiol Plant. 2026 Oct 2;178(5):e71141. doi: 10.1111/ppl.71141 (PMC13633127; doi:10.1111/ppl.71141)
Supplement: Supplementary file 1 [file PPL-178-e71141-s001.pdf]

Far-red light supplementation in tomato cultivation: A quantitative review of morphological and physiological responses

Anna Fischer[1], Irene Vänninen [2], Kristiina Himanen[1,3]

Supplementary Table S2: Parameters examining plant growth and morphology in controlled environments, number of studies and observations within those examining these parameters, detected effects of far-red light on these parameters ("+" stands for a statistically significant increase upon FR exposure, "0" for no statistically significant effect, and "-" for a statistically significant decrease), the magnitude of change in per cent, and the corresponding studies by their reference number.

**Parameter**	**Number of studies**	**Number of observations**	**Detected effects of far-red light**	**Detected effect of far-red light in % of observations**	**Magnitude of change**	**References**
Plant height	15	36	**+** (15 studies, 31 observations)<br>**0** (2 studies, 4 observations)<br>**-** (1 study, 1 observation)	**+:** 86.1%<br>**0:** 11.1%<br>**-:** 2.8%	**Median:** +36.2%<br>**Mean:** +68.8%<br>**Range:** -35% to +254.5%<br><br>N = 35 observations	**+:** 44, 36, 50, 5, 13, 72, 19, 61, 77, 59, 82, 21, 29, 46, 52<br>**0:** 50, 29<br>**-:** 19
Hypocotyl length	3	11	**+** (3 studies, 5 observations)<br>**0** (2 studies, 4 observations)<br>**-** (1 study, 2 observations)	**+:** 45.5%<br>**0:** 36.4%<br>**-:** 18.2%	**Median:** +8.6%<br>**Mean:** +7.4%<br>**Range:** -20.6% to +39.5%<br><br>N = 11 observations	**+:** 50, 28, 49<br>**0:** 50, 28<br>**-:** 28
Leaf area	9	23	**+** (8 studies, 12 observations)<br>**0** (4 studies, 7 observations)<br>**-** (2 studies, 4 observations)	**+:** 52.2%<br>**0:** 30.4%<br>**-:** 17.4%	**Median:** +12.4%<br>**Mean:** +19.6%<br>**Range:** -27.3% to +128.6%<br><br>N = 22 observations	**+:** 13, 72, 61, 77, 59, 29, 46, 52<br>**0:** 44, 61, 77, 29<br>**-:** 61, 29
Fresh weight	6	16	**+** (5 studies, 9 observations)<br>**0** (0 studies, 0 observations)	**+:** 56.3%<br>**0:** 0%<br>**-:** 43.8%	**Median:** +23.1%<br>**Mean:** +16.2%	**+:** 44, 5, 72, 59, 21<br>**0:** -<br>**-:** 5, 28

			**-** (2 studies, 7 observations)		**Range:** -77.8% to +133.3%<br><br>N = 16 observations	
Dry weight	15	43	**+** (10 studies, 21 observations)<br>**0** (8 studies, 12 observations)<br>**-** (5 studies, 10 observations)	**+:** 48.8%<br>**0:** 27.9%<br>**-:** 23.3%	**Median:** +22.2%<br>**Mean:** +11.2%<br>**Range:** -75.5% to +88.9%<br><br>N = 41 observations	**+:** 36, 5, 13, 72, 61, 77, 30, 59, 52, 65<br>**0:** 36, 50, 28, 13, 61, 82, 84<br>**-:** 36, 5, 28, 65
Stem diameter	5	15	**+** (1 study, 1 observation)<br>**0** (4 studies, 7 observations)<br>**-** (3 studies, 7 observations)	**+:** 6.7%<br>**0:** 46.7%<br>**-:** 46.7%	**Median:** -6.4%<br>**Mean:** -12.3%<br>**Range:** -38.3% to +9.7%<br><br>N = 13 observations	**+:** 72<br>**0:** 5, 50, 28, 19<br>**-:** 5, 28, 19
Leaf number	5	11	**+** (2 studies, 2 observations)<br>**0** (4 studies, 9 observations)<br>**-** (0 studies, 0 observations)	**+:** 18.2%<br>**0:** 81.8%<br>**-:** 0%	**Median:** +12.9%<br>**Mean:** +23.1%<br>**Range:** +3.2% to +63.3%<br><br>N = 4 observations	**+:** 46, 65<br>**0:** 36, 72, 59, 65<br>**-:** -
Leaf thickness	3	6	**+** (1 study, 1 observation)<br>**0** (3 studies, 5 observations)<br>**-** (0 studies, 0 observations)	**+:** 16.7%<br>**0:** 83.3%<br>**-:** 0%	**Median:** -18.3%<br>**Mean:** -18.3%<br>**Range:** -20.4% to -16.1%<br><br>N = 2 observations	**+:** 13<br>**0:** 13, 73, 65<br>**-:** -
Leaf length	2	5	**+** (1 study, 2 observations)<br>**0** (2 studies, 3 observations)<br>**-** (0 studies, 0 observations)	**+:** 40%<br>**0:** 60%<br>**-:** 0%	**Median:** +6.9%<br>**Mean:** +5.4%<br>**Range:** -10% to +13%<br><br>N = 5 observations	**+:** 50<br>**0:** 50, 19<br>**-:** -
Specific leaf area	3	9	**+** (0 studies, 0 observations)<br>**0** (3 studies, 9 observations)	**+:** 0%<br>**0:** 100%<br>**-:** 0%	**Median:** +9.1%<br>**Mean:** +8.4%<br>**Range:** -9.1% to +21.5%	**+:** -<br>**0:** 13, 61, 65<br>**-:** -

			**-** (0 studies, 0 observations)		N = 8 observations	
Shoot:root ratio	2	4	**+** (1 study, 1 observation)<br>**0** (2 studies, 3 observations)<br>**-** (0 studies, 0 observations)	**+:** 25%<br>**0:** 75%<br>**-:** 0%	**Median:** +33%<br>**Mean:** +34.8%<br>**Range:** +22.4% to +50.9%<br><br>N = 4 observations	**+:** 30<br>**0:** 77, 30<br>**-:** -

Supplementary Table S3: Parameters examining reproductive growth in controlled environments, number of studies and observations examining these parameters, detected effects of adding far-red light on these parameters ("+" stands for a statistically significant increase upon FR exposure, "0" for no statistically significant effect, and "-" for a statistically significant decrease), the magnitude of change in per cent, and the corresponding studies.

**Parameter**	**Number of studies**	**Number of observations**	**Detected effects of far-red light**	**Detected effect of far-red light in % of observations**	**Magnitude of change**	**References**
Flowering time	2	5	**+** (2 studies, 4 observations)<br>**0** (0 studies, 0 observations)<br>**-** (1 study, 1 observation)	**+:** 80%<br>**0:** 0%<br>**-:** 20%	**Median:** +26.3%<br>**Mean:** +22.9%<br>**Range:** -17.9% to +50%<br><br>N = 5 observations	**+:** 6, 65<br>**0:** -<br>**-:** 65
Ripe fruits glucose and fructose content	2	2	**+** (1 study, 1 observation)<br>**0** (1 study, 1 observation)<br>**-** (0 studies, 0 observations)	**+:** 50%<br>**0:** 50%<br>**-:** 0%	**Median:** +22.4%<br>**Mean:** +22.4%<br>**Range:** +7.7% to +37.1%<br><br>N = 2 observations	**+:** 31<br>**0:** 52<br>**-:** -

Supplementary Table S4: Parameters examining photosynthesis of tomato plants in controlled environments, number of studies and observations examining these parameters, detected effects of adding far-red light on these parameters ("+" stands for a statistically significant increase upon FR exposure, "0" for no statistically significant effect, and "-" for a statistically significant decrease), the magnitude of change in per cent, and the corresponding studies.

Parameter	Number of studies	Number of observations	Detected effects of far-red light	Detected effect of far-red light in % of observations	Magnitude of change	References
Leaf chlorophyll content	7	24	**+** (0 studies, 0 observations)<br>**0** (2 studies, 5 observations)<br>**-** (7 studies, 19 observations)	**+:** 0%<br>**0:** 20.8%<br>**-:** 79.2%	**Median:** -15.5%<br>**Mean:** -18.2%<br>**Range:** -42.9% to -3.1%<br><br>N = 24 observations	**+:** -<br>**0:** 50, 5<br>**-:** 50, 6, 5, 72, 19, 61, 59, 46
Maximum quantum yield PSII	3	9	**+** (1 study, 1 observation)<br>**0** (3 studies, 8 observations)<br>**-** (0 studies, 0 observations)	**+:** 11.1%<br>**0:** 88.9%<br>**-:** 0%	**Median:** +0.7%<br>**Mean:** +0.9%<br>**Range:** +/-0% to +1.9%<br><br>N = 3 observations	**+:** 65<br>**0:** 72, 29, 65<br>**-:** -
Effective quantum yield PSII	3	11	**+** (2 studies, 5 observations)<br>**0** (1 study, 1 observation)<br>**-** (2 studies, 5 observations)	**+:** 45.5%<br>**0:** 9.1%<br>**-:** 45.5%	**Median:** -1.7%<br>**Mean:** +1.7%<br>**Range:** -14.3% to +20.9%<br><br>N = 11 observations	**+:** 29, 65<br>**0:** 65<br>**-:** 5, 29
Net photosynthesis rate	4	9	**+** (1 study, 1 observation)<br>**0** (1 study, 1 observation)<br>**-** (2 studies, 6 observations)	**+:** 11.1%<br>**0:** 11.1%<br>**-:** 66.7%	**Median:** -17.8%<br>**Mean:** -12.6%<br>**Range:** -37.1% to +14.9%	**+:** 82, 84<br>**0:** 29<br>**-:** 72, 29

					N = 9 observations	
